# Anxiety and depression among victims of work-related upper extremity injuries

**DOI:** 10.1192/j.eurpsy.2022.777

**Published:** 2022-09-01

**Authors:** A. Haddar, I. Sellami, A. Hrairi, N. Rmadi, R. Masmoudi, K. Hammami, J. Masmoudi, M.L. Masmoudi, M. Hajjaji

**Affiliations:** 1 Hedi Chaker University Hospital of Sfax, Occupational Medecine, Sfax, Tunisie, Tunisia; 2 Hedi Chaker university hospital, Occupational Medicine, Sfax, Tunisia; 3 Hedi Chacker Hospital, Occupational Medicine, Sfax, Tunisia; 4 HEDI CHAKER hospital, Department Of Occupational Medicine, SFAX, Tunisia; 5 HEDI CHAKER hospital, Psychiatry Department, SFAX, Tunisia; 6 Hospital university of HEDI CHAKER, Psychiatry A Department, Sfax, Tunisia

**Keywords:** Depression, occupational accident, Anxiety

## Abstract

**Introduction:**

Being a victim of work-related upper extremity injuries is a source of physical damage and mental damages. Psychological distress related to this type of accident is usually underestimated.

**Objectives:**

Evaluate anxiety and depression among victims of work-related upper extremity injuries.

**Methods:**

We conducted a 10-month cross-sectional analysis on workers consulting for an Impairment Rating Evaluation after an upper extremity injury due to an occupational accident. We collected socio-professional data, characteristics and outcomes of the accident. Anxiety and depression were evaluated by the Hospital Anxiety and Depression scale. The pain was evaluated by a Visual Analogue Scale.

**Results:**

Our population consisted of 90 cases of work-related upper-extremity injuries. The mean age was 43.10 and the sex ratio 3.7. The most represented category was blue-collar workers (71.1%). Medical history of chronic diseases was reported in 23% of cases and 3.3 % had mental health antecedent. Dominant upper limb injuries were found in 62% of cases. Hand and wrist injuries were the most affected part (63%), and 33.3% had fingers’ injuries. The prevalence of anxiety and depression were 31.1% and 20% respectively. About thirty-one per cent rated their current pain greater than or equal to 8. Both anxiety and depression were positively correlated with male gender (p= 0.001, p=0.007) and shoulder injuries (p=0.001, p=0.018). Depression was correlated to fingers’ injuries and pain (p=0.002).

**Conclusions:**

The studied population present an important rate of anxiety and depression. Assessing Mental health after upper extremities injuries are necessary to prevent serious mental illness and to promote a successful return to work.

**Disclosure:**

No significant relationships.

